# No improvement in mortality among critically ill patients with carbapenems as initial empirical therapy and more detection of multi-drug resistant pathogens associated with longer use: a post hoc analysis of a prospective cohort study

**DOI:** 10.1128/spectrum.00342-24

**Published:** 2024-06-12

**Authors:** Junki Ishii, Mitsuaki Nishikimi, Liesbet De Bus, Jan De Waele, Akihiro Takaba, Akira Kuriyama, Atsuko Kobayashi, Chie Tanaka, Hideki Hashi, Hideki Hashimoto, Hiroshi Nashiki, Mami Shibata, Masafumi Kanamoto, Masashi Inoue, Satoru Hashimoto, Shinshu Katayama, Shinsuke Fujiwara, Shinya Kameda, Shunsuke Shindo, Tetsuya Komuro, Toshiomi Kawagishi, Yasumasa Kawano, Yoshihito Fujita, Yoshiko Kida, Yuya Hara, Hideki Yoshida, Shigeki Fujitani, Nobuaki Shime, on behalf of the DIANA study Japanese group

**Affiliations:** 1 Department of Emergency and Critical Care Medicine, Graduate School of Biomedical and Health Sciences, Hiroshima University, Hiroshima, Japan; 2 Department of Emergency and Critical Care Medicine, Nagoya University Graduate School of Medicine, Aichi, Japan; 3 Department of Intensive Care Medicine, Ghent University Hospital, Ghent, Belgium; 4 Department of Internal Medicine and Pediatrics, Faculty of Medicine and Health Sciences, Ghent University, Ghent, Belgium; 5 JA Hiroshima General Hospital, Hiroshima, Japan; 6 Emergency and Critical Care Center, Kurashiki Central Hospital, Okayama, Japan; 7 Takarazuka City Hospital, Hyogo, Japan; 8 Nippon Medical School Tama Nagayama Hospital, Tokyo, Japan; 9 Tokyo Bay Urayasu Ichikawa Medical Center, Chiba, Japan; 10 Hitachi General Hospital, Ibaraki, Japan; 11 Iwate Prefectural Central Hospital, Iwate, Japan; 12 Department of Emergency and Critical Care Medicine, Wakayama Medical University Hospital, Wakayama, Japan; 13 Department of Anesthesiology, Gunma Prefectural Cardiovascular Center, , Gunma, Japan; 14 Department of Anesthesiology, Nagoya City University Hospital, Aichi, Japan; 15 Non-Profit Organization ICU Collaboration Network (ICON), Tokyo, Japan; 16 Division of Intensive Care, Department of Anesthesiology and Intensive Care Medicine, Jichi Medical University School of Medicine, Tochigi, Japan; 17 National Hospital Organization Ureshino Medical Center, Saga, Japan; 18 Jikei University School of Medicine Hospital, Tokyo, Japan; 19 Omori Red Cross Hospital, Tokyo, Japan; 20 Department of General Internal Medicine, TMG Muneoka Central Hospital, Saitama, Japan; 21 Toyama University Hospital, Toyama, Japan; 22 Fukuoka University Hospital, Fukuoka, Japan; 23 Aichi Medical University Hospital, Aichi, Japan; 24 Yodogawa Christian Hospital, Osaka, Japan; 25 Department of Emergency and Critical Care Medicine, St. Marianna University School of Medicine, Kanagawa, Japan; Mayo Foundation for Medical Education and Research, Rochester, Minnesota, USA

**Keywords:** initial empirical therapy, critically ill, infectious disease, carbapenem, multidrug resistance, intensive care unit

## Abstract

Whether empirical therapy with carbapenems positively affects the outcomes of critically ill patients with bacterial infections remains unclear. This study aimed to investigate whether the use of carbapenems as the initial antimicrobial administration reduces mortality and whether the duration of carbapenem use affects the detection of multidrug-resistant (MDR) pathogens. This was a post hoc analysis of data acquired from Japanese participating sites from a multicenter, prospective observational study [Determinants of Antimicrobial Use and De-escalation in Critical Care (DIANA study)]. A total of 268 adult patients with clinically suspected or confirmed bacterial infections from 31 Japanese intensive care units (ICUs) were analyzed. The patients were divided into two groups: patients who were administered carbapenems as initial antimicrobials (initial carbapenem group, *n* = 99) and those who were not administered carbapenems (initial non-carbapenem group, *n* = 169). The primary outcomes were mortality at day 28 and detection of MDR pathogens. Multivariate logistic regression analysis revealed that mortality at day 28 did not differ between the two groups [18 (18%) vs 27 (16%), respectively; odds ratio: 1.25 (95% confidence interval (CI): 0.59–2.65), *P* = 0.564]. The subdistribution hazard ratio for detecting MDR pathogens on day 28 per additional day of carbapenem use is 1.08 (95% CI: 1.05–1.13, *P* < 0.001 using the Fine-Gray model with death regarded as a competing event). In conclusion, in-hospital mortality was similar between the groups, and a longer duration of carbapenem use as the initial antimicrobial therapy resulted in a higher risk of detection of new MDR pathogens.

**IMPORTANCE:**

We found no statistical difference in mortality with the empirical use of carbapenems as initial antimicrobial therapy among critically ill patients with bacterial infections. Our study revealed a lower proportion of inappropriate initial antimicrobial administrations than those reported in previous studies. This result suggests the importance of appropriate risk assessment for the involvement of multidrug-resistant (MDR) pathogens and the selection of suitable antibiotics based on risk. To the best of our knowledge, this study is the first to demonstrate that a longer duration of carbapenem use as initial therapy is associated with a higher risk of subsequent detection of MDR pathogens. This finding underscores the importance of efforts to minimize the duration of carbapenem use as initial antimicrobial therapy when it is necessary.

## INTRODUCTION

Determining the appropriate antimicrobials to be used as the initial empirical therapy in critically ill patients with bacterial infections remains challenging, especially considering that the initial administration of antibiotics is time-sensitive in severely ill patients to achieve better outcomes (1, 2). Previous studies have revealed that inappropriate initial antimicrobial administration is associated with higher mortality (3–5); therefore, physicians tend to administer broad-spectrum antibiotics as initial therapy to avoid inappropriate administrations. The Determinants of Antimicrobial Use and De-escalation in Critical Care (DIANA) study, a multicenter international observational cohort study investigating critically ill adult patients receiving empirical antimicrobial therapy for suspected or confirmed bacterial infections in the intensive care unit (ICU), revealed that carbapenems are some of the most widely administered antimicrobials as initial therapy [389/1,495 (26%) patients were administered carbapenems as empirical antimicrobial therapy] (6). However, it remains unclear whether the empirical use of carbapenems improves mortality rates.

Questions regarding whether carbapenems as initial empirical therapy lead to improvement in mortality, have arisen for several reasons. First, various studies on the treatment of bacterial infections and risk assessment of involvement of multidrug-resistant (MDR) pathogens have been expanding globally (7–13). Several recent studies have identified the risk for MDR pathogens, helping us select narrow antibiotics more accurately in cases with a low risk for MDR pathogens (7–13). This can potentially counteract the benefits of using carbapenems empirically to avoid inappropriate therapy. Second, previous studies have reported that extended-spectrum beta-lactamase-producing *Enterobacteriaceae*, a major reason for the use of carbapenems as initial empirical therapy (14), are frequently susceptible to other antibiotics such as specific beta-lactam/beta-lactamase inhibitors (15, 16), although a few studies have indicated the possibility of the superiority of carbapenems over other antibiotics (17, 18). Several studies have implied that carbapenem-sparing regimens as empirical therapy for various infectious diseases show non-inferiority to carbapenems (15, 16, 19–24). However, the effect is unknown in an integrated cohort of critically ill patients with bacterial infection in the ICU setting.

The subsequent detection of MDR pathogens is another concern related to carbapenem use. A previous study revealed that the use of broad-spectrum antimicrobials, including carbapenems as initial empirical therapy for more than 72 h was associated with the detection of new MDR pathogens (25). However, no studies are focusing on carbapenems, which are broad-spectrum antimicrobials that may be considered to have the greatest impact on the emergence of MDR pathogens, except for a single-center study (26).

We hypothesized that the use of carbapenems as initial antimicrobial therapy would not improve outcomes and that the extended duration of the initial empirical therapy with carbapenems was a risk factor for the subsequent detection of MDR pathogens. This study was a post hoc analysis aimed at investigating whether the use of carbapenems as the initial administration can improve mortality on day 28 and whether the duration of use increases the subsequent detection of MDR pathogens.

## RESULTS

**Fig 1:**
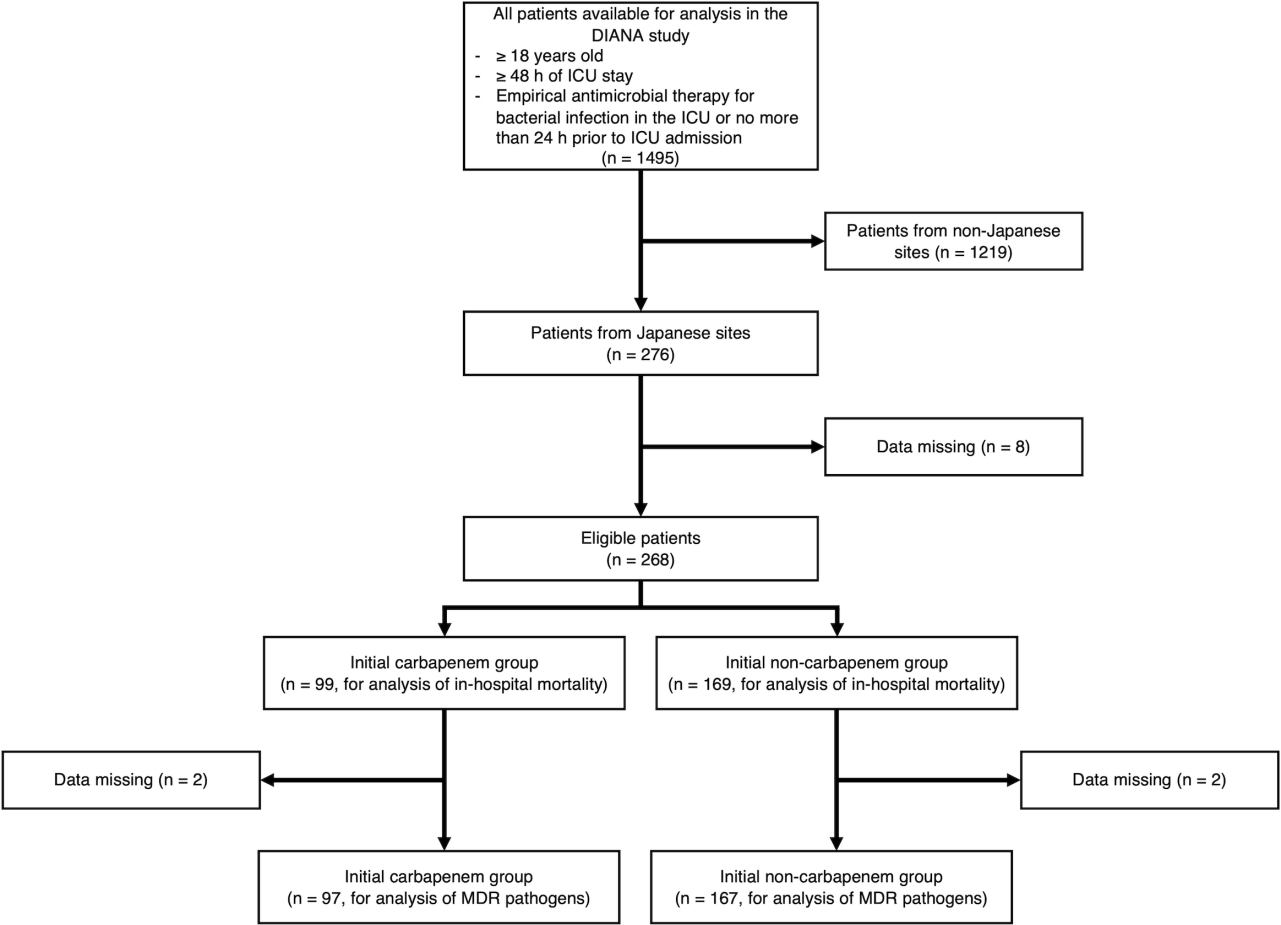
Patient flowchart.

Figure 1 illustrates the patient flowchart of the study. Among the 1,495 patients registered in the DIANA study, 276 from 31 Japanese ICUs were eligible for inclusion in this study. Eight patients were excluded according to our preset criteria because of missing values required for the analyses. As a result, the remaining 268 patients were analyzed. Among them, 99 (37%) were administered carbapenems as initial empirical therapy, whereas 169 (63%) were administered other antibiotics. A list of initial antimicrobial therapy in the initial non-carbapenem group is shown in Table S1.

The baseline characteristics of the 268 patients are summarized in Table 1. There were no statistically significant differences between the two groups, except for antimicrobial exposure from hospitalization to study inclusion, the sources of infection, and bloodstream infection. In the initial carbapenem group, the median duration of carbapenem use as initial empirical therapy was 6 days (interquartile range: 3–9). Eighteen patients (18%) in the initial carbapenem group and 27 patients (16%) in the initial non-carbapenem group had died in the hospital by day 28. The odds ratio (OR) for the use of carbapenems for in-hospital mortality by day 28 was 1.25 (95% confidence interval (CI): 0.59–2.65) in the multivariate analysis (Table 2).

**TABLE 1:** Patient characteristics

Variable*^a^*	Overall cohort (*n* = 268)	Initial carbapenem (*n* = 99)	Initial non-carbapenem (*n* = 169)
Age (yr)*^b^*	73 (60–80)	70 (62–80)	74 (58–81)
Male sex, no. (%)	151 (56)	55 (56)	96 (57)
APACHE II on ICU admission*^b^*	21 (15–26)	21 (16–26)	20 (15–27)
Abx exposure before inclusion, no. (%)	123 (46)	60 (61)	63 (37)
Comorbidity			
Chronic pulmonary disease, no. (%)	28 (10)	12 (12)	16 (9)
Chronic hepatic disease, no. (%)	15 (6)	6 (6)	9 (5)
Chronic renal disease, no. (%)	32 (12)	13 (13)	19 (11)
Diabetes mellitus, no. (%)	57 (21)	25 (25)	32 (19)
Cardiovascular disease, no. (%)	61 (23)	18 (18)	43 (25)
Solid tumor, no. (%)	43 (16)	14 (14)	29 (17)
Hematologic malignancy, no. (%)	9 (3)	6 (6)	3 (2)
Cerebrovascular disease, no. (%)	31 (12)	14 (14)	17 (10)
Healthcare exposure*^c^*, no. (%)	115 (43)	43 (43)	72 (43)
Immunosuppression status*^d^*, no. (%)	37 (14)	16 (16)	21 (12)
Colonization with MDR pathogens prior to initiation of empirical antimicrobials*^e^*, no. (%)	11 (4)	6 (6)	5 (3)
Source of infection			
Abdominal, no. (%)	66 (25)	40 (40)	26 (15)
Respiratory, no. (%)	93 (35)	17 (17)	76 (45)
Urogenital, no. (%)	24 (9)	8 (8)	16 (9)
Others, no. (%)	93 (35)	34 (34)	59 (35)
Bloodstream infection, no. (%)	68 (25)	37 (37)	31 (18)
Microbiologically documented infection, no. (%)	137 (51)	59 (60)	78 (46)
Septic shock, no. (%)	78 (29)	35 (35)	43 (25)

^*a*^ APACHE, Acute Physiology and Chronic Health Evaluation; ICU, intensive care unit; Abx, antibiotics; HIV, human immunodeficiency virus; AIDS, acquired immunodeficiency syndrome; MDR, multidrug-resistant.

^*b*^ Data are presented as median (interquartile range).

^*c*^ Hospitalization for ≥2 days in the 12 months preceding study inclusion, antimicrobial exposure in the previous 3 months preceding study inclusion, resident in a nursing home or long-term care facility, receiving chronic hemodialysis or receiving invasive procedures (at home or in an outpatient clinic) in the previous 30 days preceding study inclusion.

^*d*^ Congenital immunodeficiency, neutropenia (absolute neutrophil count <1,000 neutrophils/μL), patient receiving corticosteroid treatment (prednisolone or equivalent >0.5 mg/kg/day for >3 months preceding study inclusion), solid organ transplant patient receiving immunosuppressive treatment, bone marrow transplant patient receiving immunosuppressive treatment, administration of chemotherapy within 1 year preceding study inclusion, administration of radiotherapy within 1 year preceding inclusion, patient with autoimmune disease receiving immunosuppressive treatment, or with HIV or AIDS.

^*e*^ Defined as the detection of MDR pathogens presumed to be present upon the ICU admission. This included those detected within 1 year prior to study inclusion, and those not present on ICU admission and detected before day 2 (day 0 was considered the start date of the empirical antimicrobial therapy).

**TABLE 2:** Odds ratios and *P* values of mortality at day 28 and ICU mortality

Outcome*^b^*	Initial carbapenem (*n* = 99)	Initial non-carbapenem (*n* = 169)	Univariate		Multivariate*^a^*	
			Odds ratio (95% CI)	*P* value	Odds ratio (95% CI)	*P* value
Mortality at day 28, no. (%)	18 (18)	27 (16)	1.17 (0.61–2.25)	0.735	1.25 (0.59–2.65)	0.564
ICU mortality, no. (%)	11 (11)	17 (10)	1.12 (0.50–2.49)	0.837	1.80 (0.71–4.56)	0.216

^*a*^ APACHE II score on ICU admission, antimicrobial exposure between hospitalization and the day of inclusion, and source of infection were used for multivariate logistic regression analysis.

^*b*^ ICU, intensive care unit; CI, confidence interval; APACHE, Acute Physiology and Chronic Health Evaluation.

There was no significant difference regarding secondary outcomes between the two groups. Eleven patients (11%) in the initial carbapenem group and 17 patients (10%) in the initial non-carbapenem group died in the ICU. The odds ratio for ICU mortality by day 28 was 1.80 (0.71–4.56) in the multivariate analysis (Table 2). The inappropriate initial antimicrobial administration was 6 (6%) in the initial carbapenem group, and 7 (4%) in the initial non-carbapenem group (*P* = 0.559). The number of days spent in the ICU and hospital following the onset of infection under study [measured from inclusion (day 0) to day 28 and assessed in subgroups of ICU survivors (*n* = 240) and patients alive at day 28 (*n* = 223)] was not statistically different between the initial carbapenem and initial non-carbapenem groups [median (interquartile range) days in the ICU: 7 (4–16) vs 7 (4–18), *P* = 0.675; days in the hospital: 28 (20–28) vs 28 (19–28), *P* = 0.651]. Similarly, the length of stay (days) in the ICU and hospital on day 28 was not statistically different between the two groups {ICU [median (interquartile range)]: 7 (3–17) vs 7 (4–20), *P* = 0.262; hospital: 28 (22–30) vs 28 (21–30), *P* = 0.985}.

MDR pathogens were newly detected in 11 patients (11%) in the initial carbapenem group and 13 patients (8%) in the initial non-carbapenem group (*P* = 0.380). Two hundred and sixty-four patients were analyzed to evaluate the association between the detection of MDR pathogens and the duration of carbapenem use as the initial antimicrobial administration. Four patients were excluded owing to missing data regarding the detection of MDR pathogens. The association between the detection of MDR pathogens and the duration of use of carbapenems was statistically significant; subdistribution hazard ratios (sHRs) for detecting MDR pathogens according to the duration of carbapenem use were 1.08 (95% CI: 1.05–1.13) in the multivariate analysis (Table 3). A list of newly detected MDR pathogens is shown in Table S2.

**TABLE 3:** Subdistribution hazard ratios and *P* values for detecting MDR pathogens according to the duration of carbapenem use as the initial antimicrobial administration*^a^*

	Univariate*^b^*		Multivariate*^c^*	
	sHR (95% CI) for detection of MDR	*P* value	sHR (95% CI) for detection of MDR	*P* value
Carbapenem use as initial antimicrobial administration (per additional day of carbapenem use)	1.08 (1.05–1.11)	<0.001	1.08 (1.05–1.13)	<0.001

^*a*^ MDR, multidrug-resistant; sHR, subdistribution hazard ratio; CI, confidence interval; APACHE, Acute Physiology and Chronic Health Evaluation; ICU, intensive care unit.

^*b*^ Patients (*n* = 264) without missing data were analyzed.

^*c*^ APACHE II score on ICU admission, antimicrobial exposure between hospitalization and the day of inclusion, and source of infection were used for multivariate analysis.

The subgroup analysis of patients treated with monotherapy (*n* = 179 for mortality at day 28 and ICU mortality; *n* = 177 for the detection of new MDR pathogens) showed results consistent with our main analysis (Tables S3 and S4).

## DISCUSSION

In this post hoc analysis of a prospective observational study of ICU patients with suspected or confirmed bacterial infections, we found no statistical difference in mortality by empirical use of carbapenems as initial antimicrobial therapy. We also demonstrated that a longer duration of carbapenem use resulted in a higher risk of emergence of new MDR pathogens.

One of the most common reasons for the use of carbapenems is to avoid inappropriate initial antimicrobial administration, which means that causative pathogens were *in vitro* not susceptible, considering that the treatment for patients with severe infections is time-sensitive (1, 2). However, the proportions of inappropriate initial antimicrobial administration in our study were 6% in the initial carbapenem group and 4% in the initial non-carbapenem group. These proportions were much lower than those reported in previous studies. One systematic review and meta-analysis reported that the overall proportion of inappropriate administration was 14.1%–78.9% (27). We assume that this discrepancy may be explained by recent improvements in the quality of treatment and those reflecting the guidelines for severe infections including those in Japan, such as enhanced clinical skills for estimating causative microorganisms and more precise risk assessment for MDR pathogens (7–13, 28), although further studies are required. The lack of significant difference in the mortality between those who were not administered carbapenems and those with carbapenem use in our study, which was similar to the recent study (29), may be associated with this low proportion of inappropriate initial antimicrobial administration in both groups.

We emphasize that our study does not imply that carbapenems are not required in actual clinical practice. For patients at high risk of MDR, we should not hesitate to use broad-spectrum antibiotics, including carbapenems (17, 18). A previous study reported that the unnecessary use of broad-spectrum antibiotics and inappropriate use of narrow-spectrum antibiotics were associated with poor patient outcomes (30). Although we did not evaluate whether the use of carbapenems as initial therapy in our study was necessary or too broad, we believe that our study highlights the importance of appropriate risk assessment for MDR pathogens and the selection of “appropriate” antibiotics according to risk. Further study is warranted from this perspective.

Detection of MDR pathogens, regardless of colonization or infection, is associated with poor patient outcomes (31–33). Although a previous study investigated the association between the duration of carbapenem use and subsequent infection with MDR pathogens (26), no study has investigated the association between the duration of carbapenem use as initial empirical therapy for critically ill patients with infections and the subsequent detection of MDR pathogens, regardless of colonization or infection. To the best of our knowledge, our study is the first to show that a longer duration of carbapenem use as initial therapy increases the rate of subsequent detection of MDR pathogens. Our findings suggest that care should be taken regarding the duration of carbapenem exposure when choosing initial empirical therapy and treatment should be de-escalated to more narrow-spectrum antibiotics as soon as possible if other antibiotics could be effective for the patient.

Our study had several limitations. First, because this was a post hoc analysis of a prospective observational study, a randomized controlled trial with sufficient participants is required to confirm the non-superiority of carbapenems as an initial empirical therapy in a well-selected patient population. Second, this study only included data obtained from participating hospitals in Japan. We need to confirm whether our findings are consistent with those of other countries, although our treatment strategy follows the guidelines for severe infections, including the Japanese national guidelines for sepsis, which are not significantly different from those of other countries (34). In addition, the proportion of resistance in Japan is not far from the majority of those reported worldwide (35). Third, we could not assess the effect of antibiotic duration on detecting new MDR pathogens because of multicollinearity. Therefore, as future studies, investigating the effects of overall exposure to antimicrobial agents in critically ill patients with bacterial infections would be beneficial.

In conclusion, in-hospital mortality was similar between critically ill patients who were administered carbapenems as initial empirical antimicrobials and those who were treated with other antibiotics, with a longer duration of carbapenem use resulting in a higher risk of new detection of MDR pathogens.

## MATERIALS AND METHODS

### Study design

This was a post hoc analysis of data obtained from Japanese participants of a prospective, multicenter international observational study (DIANA study), which analyzed 1,495 critically ill adult patients receiving empirical antimicrobial therapy for suspected or confirmed bacterial infections at 152 ICUs in 28 countries from October 2016 to May 2018 (6). Patients were included in the DIANA study if they were 18 years or older, admitted to an ICU with an anticipated need for at least 48 h of ICU support, and were treated with empirical antimicrobial therapy in the ICU, or no more than 24 h prior to ICU admission to treat a suspected or confirmed community-, healthcare-, hospital-, or ICU-acquired bacterial infection. Patients were excluded if they were already included in the DIANA study or had insufficient data on infection and/or microbiology. In this post hoc analysis, patients from 31 Japanese ICUs were included in the DIANA study. Patients with missing values for the variables required for analysis were excluded. The patients were divided into two groups: one group was administered carbapenems as initial empirical antimicrobial therapy (initial carbapenem group) and the other group without carbapenems (initial non-carbapenem group).

The study was conducted by the Declaration of Helsinki and was approved by the Institutional Review Board of Hiroshima University, which waived the requirement for informed consent to ensure participant anonymity, as stipulated in the Japanese government guidelines (approval no. E2021-2721).

### Data set

In the original DIANA study, data on patients, infections (including information on MDR pathogens), antimicrobial treatment, and outcomes were collected from the day of study inclusion (day 0), defined as the start date of empirical antimicrobial therapy, to day 28 following initiation.

### Definitions

Initial empirical antimicrobial administration was defined as the administration when the causative pathogen and susceptibility pattern were unidentified at the time of initiation of antimicrobial therapy. Day 0 was considered the start date of the empirical antimicrobial therapy. The detection of MDR pathogens was defined as detection between days 2 and 28 and absence before day 2, and the positive sample sites were categorized as follows: nose swab, throat swab, respiratory tract samples, urine samples, rectal swab/fecal samples, blood culture, perioperative samples, and others. Colonization with MDR pathogens before the initiation of empirical antimicrobials was defined as the detection of MDR pathogens presumed to be present upon ICU admission. It included those detected within 1 year prior to study inclusion, and those not present on ICU admission and detected before day 2. Multidrug resistance was defined as a pathogen producing extended-spectrum beta-lactamase or carbapenemase, *Stenotrophomonas maltophilia*, methicillin-resistant *Staphylococcus aureus*, vancomycin-resistant *Enterococcus* sp., or a pathogen resistant to three or more antimicrobial classes, according to the publication of Magiorakos et al. (36). Empirical therapy was considered inappropriate when a causative pathogen resistant to the initial agent(s) was present that leading to the addition or replacement of the empirical antimicrobial administration.

### Outcome measurement

The primary outcomes were in-hospital mortality and detection of new MDR pathogens by day 28. Secondary outcomes were ICU mortality by day 28, inappropriate empirical antimicrobial administrations, and number of days in ICU and hospital following the onset of infection under study [measured from inclusion (day 0) to day 28, and assessed in subgroups of ICU survivors and patients alive at day 28, respectively]. Mortality was compared between the two groups. The risk of new detection of MDR pathogens was evaluated according to the duration (days) of use of carbapenems as the initial antimicrobial therapy; the duration was regarded as zero for the initial non-carbapenem group. Subgroup analyses were performed for patients treated with monotherapy.

### Statistical analysis

A complete case analysis was conducted. Continuous variables were expressed as medians (interquartile range: 25–75), and categorical variables were expressed as proportions (%). Fisher’s exact test was used to compare categorical variables between the groups. The Mann–Whitney U-test was used to compare the continuous variables. In the multivariate logistic regression analysis, the adjustment factors were chosen beforehand according to clinical aspects: Acute Physiology and Chronic Health Evaluation II (APACHE II) scores on ICU admission, antimicrobial exposure between hospitalization and the day of inclusion, and the source of infection (abdominal, respiratory, urogenital, or others) (37–42). To determine the risk of detection of MDR pathogens according to the duration (days) of use of carbapenems as the initial antimicrobial therapy, the Fine-Gray model, with death as a competing event, was used. The adjustment factor was the same as that used in multivariate logistic regression analysis.

All reported *P* values were two-sided, and statistical significance was set at *P* < 0.05. All analyses were performed using R, version 4.2.2 (Vienna University of Economics and Business, Vienna, Austria) and JMP Pro 16 software (SAS Institute, Cary, NC, USA).

## Data Availability

Data are available upon reasonable request with the permission of participating facilities.

## SUPPLEMENTAL MATERIAL

Supplemental tables spectrum.00342-24-s0001.docx Tables S1-S4.

ASM does not own the copyrights to Supplemental Material that may be linked to, or accessed through, an article. The authors have granted ASM a non-exclusive, world-wide license to publish the Supplemental Material files. Please contact the corresponding author directly for reuse.
