# Supplementary material for: No improvement in mortality among critically ill patients with carbapenems as initial empirical therapy and more detection of multi-drug resistant pathogens associated with longer use: a post hoc analysis of a prospective cohort study
Source: Microbiol Spectr. 2024 Jun 12;12(7):e00342-24. doi: 10.1128/spectrum.00342-24 (PMC11218456; doi:10.1128/spectrum.00342-24)
Supplement: Supplemental tables — Tables S1-S4. [file spectrum.00342-24-s0001.docx]

**TABLE S1** Empirical antimicrobial therapy among patients who were not administered carbapenems as initial therapy

|  | **Treatment**  **Total n = 169** |
| --- | --- |
| Antipseudomonal penicillins + β-lactamase inhibitor, no. (%) | 72 (32.3) |
| Penicillins + β-lactamase inhibitor, no. (%) | 41 (18.4) |
| Glycopeptides, no. (%) | 26 (11.7) |
| Third-generation cephalosporins, no. (%) | 25 (11.2) |
| First-generation cephalosporins, no. (%) | 15 (6.7) |
| Macrolides, no. (%) | 8 (3.6) |
| Second-generation cephalosporins, no. (%) | 7 (3.1) |
| Fluoroquinolones, no. (%) | 7 (3.1) |
| Penicillins, no. (%) | 5 (2.2) |
| Fourth-generation cephalosporins, no. (%) | 4 (1.8) |
| Lincosamides, no. (%) | 3 (1.3) |
| Folate pathway inhibitor, no. (%) | 2 (0.9) |
| Lipopeptides, no. (%) | 2 (0.9) |
| Nitroimidazoles, no. (%) | 2 (0.9) |
| Aminoglycosides, no. (%) | 1 (0.4) |
| Oxazolidinones, no. (%) | 1 (0.4) |
| Tetracyclines, no. (%) | 1 (0.4) |
| Other, no. (%) | 1 (0.4) |

**TABLE S2** Newly detected MDR pathogens*^ab^*

|  | **Initial carbapenem**  **(n =11/99 [11%])** | **Initial non-carbapenem**  **(n = 13/169 [8%])** |
| --- | --- | --- |
|  | n/14 pathogens (%) | n/14 pathogens (%) |
| **Aerobic gram-positive bacteria** |  |  |
| *Staphylococcus sp.* |  |  |
| ·       *Staphylococcus aureus* | 5 (36) | 4 (29) |
| *Enterococcus sp.* | 0 (0) | 1 (7) |
|  |  |  |
| **Aerobic gram-negative pathogens** |  |  |
| Enterobacterales |  |  |
| ·       *Enterobacter sp.* | 1 (7) | 0 (0) |
| ·       *Escherichia sp.* | 3 (21) | 5 (36) |
| ·       *Proteus sp.* | 0 (0) | 1 (7) |
| ·       *Serratia sp.* | 0 (0) | 1 (7) |
| ·       *Other Enterobacteriaceae sp.* | 0 (0) | 1 (7) |
| Non-fermenting bacteria |  |  |
| ·       *Pseudomonas aeruginosa* | 1 (7) | 0 (0) |
| ·       Other Non-fermenting bacteria*^c^* | 4 (29) | 1 (7) |

*^a^*MDR, multidrug-resistant.

*^b^*Multidrug resistance was defined as a pathogen producing extended-spectrum beta-lactamase (ESBL) or carbapenemase, *Stenotrophomonas maltophilia*, methicillin-resistant *Staphylococcus aureus*, vancomycin-resistant *Enterococcus* sp., or a pathogen resistant to three or more antimicrobial classes according to the publication of Magiorakos et al. (1).

*^c^Achromobacter sp., Acinetobacter sp., Burkholderia sp., Elizabethkingia sp., Pseudomonas* non-aeruginosa*, Sphingomonas sp., Stenotrophomonas sp.*

Reference

1. Magiorakos AP, Srinivasan A, Carey RB, Carmeli Y, Falagas ME, Giske CG, Harbarth S, Hindler JF, Kahlmeter G, Olsson-Liljequist B, Paterson DL, Rice LB, Stelling J, Struelens MJ, Vatopoulos A, Weber JT, Monnet DL. 2012. Multidrug-resistant, extensively drug-resistant, and pandrug-resistant bacteria: an international expert proposal for interim standard definitions for acquired resistance. Clin Microbiol Infect 18:268-81.

**TABLE S3** Odds ratios and *P* values of mortality at day 28 and ICU mortality among the patients with monotherapy

|  |  |  | **Univariate** | |  | **Multivariate*^a^*** | |
| --- | --- | --- | --- | --- | --- | --- | --- |
| **Outcome*^b^*** | **Initial carbapenem (n = 59)** | **Initial non-carbapenem (n = 120)** | **Odds ratio (95% CI)** | ***P* value** |  | **Odds ratio**  **(95% CI)** | ***P* value** |
| Mortality at day 28, no. (%) | 7 (12) | 17 (14) | 0.81 (0.32–2.09) | 0.816 |  | 0.76 (0.22–2.67) | 0.672 |
| ICU mortality, no. (%) | 3 (5) | 12 (10) | 0.48 (0.13–1.78) | 0.391 |  | 1.10 (0.24–5.11) | 0.902 |

*^a^*APACHE II score on ICU admission, antimicrobial exposure between hospitalization and the day of inclusion, and source of infection were used for multivariate logistic regression analysis.

*^b^*ICU, intensive care unit; CI, confidence interval; APACHE, Acute Physiology and Chronic Health Evaluation.

**TABLE S4** Subdistribution hazard ratios and *P* values for detecting MDR pathogens according to the duration of carbapenem use as the initial antimicrobial administration among patients receiving monotherapy*^a^*

|  | **Univariate*^b^*** | |  | **Multivariate*^c^*** | |
| --- | --- | --- | --- | --- | --- |
|  | **sHR (95% CI) for detection of MDR** | ***P* value** |  | **sHR (95% CI) for detection of MDR** | ***P* value** |
| Carbapenem use as initial antimicrobial administration (per additional day of carbapenem use) | 1.08 (1.05–1.11) | <0.001 |  | 1.08 (1.04–1.11) | <0.001 |

*^a^*MDR, multidrug resistance; sHR, subdistribution hazard ratio; CI, confidence interval; APACHE, Acute Physiology and Chronic Health Evaluation; ICU, intensive care unit.

*^b^*Patients who were administered a single antimicrobial agent and had no missing data (n = 177) were analyzed.

*^c^*APACHEⅡ score on ICU admission, antimicrobial exposure between hospitalization and the day of inclusion, and source of infection were used for multivariate analysis.
